# Empowering Women’s Health: Examining the Impact of Human Papillomavirus (HPV) Vaccination on Cervical Cancer Treatment and Beyond

**DOI:** 10.7759/cureus.67287

**Published:** 2024-08-20

**Authors:** Mohan Babu, Anjali Thakur, Mukthavaram Sravyasri, Gagan Gunjan, Suneeth Shetty, Kinnor Das, Ishan Y Pandya, Lavanya

**Affiliations:** 1 Department of Radiation Oncology, Regional Institute of Medical Sciences (RIMS), Manipur, IND; 2 Department of Repertory, University College of Homoeopathy, Kekri, IND; 3 Department of Medicine, Dr. NTR University of Health Sciences, Vijayawada, IND; 4 Department of General Medicine, Rajendra Institute of Medical Sciences, Ranchi, IND; 5 Department of Conservative Dentistry and Endodontics, JSS Dental College and Hospital, JSS Academy of Higher Education and Research (JSSAHER), Mysore, IND; 6 Department of Dermatology, Apollo Clinic, Silchar, IND; 7 Department of Biochemistry, Clonaexon Education and Research Institute, Gandhinagar, IND; 8 Department of Nursing, Gitam Institute of Nursing, Gitam University, Hyderabad, IND

**Keywords:** women's health, societal impact, public health, preventive healthcare, immunization programs, cervical cancer screening, cervical cancer

## Abstract

This study aims to identify the change in the health status of women, particularly in cervical cancer treatment through HPV vaccination. Thus, the research aims to measure the reduction in the incidence of cervical cancer in vaccinated women and evaluate the impact of HPV vaccination on the overall health and well-being of women treated for cervical cancer. The paper uses a research approach that involves reviewing the literature, analysing epidemiological data, and assessing the impact of the vaccination program. Major observations suggest that many developed countries’ campaigns have reduced cervical cancer and enhanced treatment. Further, the study also addresses some additional effects of the intervention, both health-related with an emphasis on the decrease in healthcare costs and an enhancement of the quality of life among women, and social with a focus on the changes in women’s status as a result of vaccination. The research also focusses on the community and economic points of view on HPV vaccination programs, its problems and opportunities regarding socio-economic factors, cultural disparities, and healthcare systems. This study implies that working on those barriers by implementing effective interventions, increasing awareness, and demanding relevant changes in policies could improve vaccination levels as well as outcomes. Hence, this research supports HPV vaccination as vital to the future health status of women. Through the use of survey data and the adoption of a public health perspective, the study can fill existing gaps in the literature on preventive interventions and cervical malignancies and consequently contribute to the enhancement of women’s health, particularly in developing countries.

## Introduction and background

The global landscape of women's health has witnessed a transformative shift with the introduction and implementation of human papillomavirus (HPV) vaccination programs. This research paper delves into the multifaceted impact of HPV vaccination, particularly focussing on its role in cervical cancer treatment and beyond. The investigation begins with an exploration of the worldwide efforts to implement HPV vaccination programs, shedding light on the varying degrees of success and challenges encountered by different countries in achieving high vaccination coverage.

The implementation of HPV vaccination programs on a global scale has been a pivotal strategy in the fight against cervical cancer. As numerous countries have embraced this preventive measure, it is essential to assess the effectiveness of these initiatives. According to recent studies, countries such as Australia and the United Kingdom have demonstrated significant success in achieving high HPV vaccination coverage rates among their target populations [1]. However, challenges persist in various regions, reflecting the complex interplay of socio-economic factors, cultural considerations, and healthcare infrastructure.

The successes and challenges faced by countries in achieving high HPV vaccination coverage are closely tied to socio-economic factors. Countries with robust healthcare systems and sufficient resources have generally fared better in implementing and sustaining vaccination programs. On the other hand, resource-poor nations may struggle due to financial constraints, hindering the widespread distribution of vaccines [2]. These discrepancies highlight the importance of addressing economic disparities to ensure equitable access to HPV vaccination globally.

Cultural considerations also play a crucial role in shaping the success of HPV vaccination initiatives. The culture that people hold and develop in their societies determines the perception they hold towards vaccines, culminating in the level of compliance observed in societies. For instance, belief patterns such as those concerning gender and sexual health may affect people’s decision to engage in vaccination campaigns. These cultural factors therefore have to be understood and dealt with when planning for the most suitable ways and angles to promote the various vaccination campaigns. For instance, cultural barriers such as the cultural perception of male and female roles and the social taboos regarding sexual health education and promotion lead to a decrease in people’s compliance with vaccination programs; it is, therefore, relevant to take cognisance of such cultural barriers to design vaccination campaigns for targeted groups [3].

Furthermore, the impact of healthcare infrastructure on the success of HPV vaccination initiatives cannot be overstated. A well-established healthcare system facilitates the effective distribution of vaccines, ensuring that they reach the target demographic. In contrast, countries facing infrastructural challenges, such as inadequate healthcare facilities and insufficient personnel, may struggle to implement and sustain vaccination programs [4]. Strengthening healthcare infrastructure emerges as a critical step in enhancing the overall success of HPV vaccination efforts.

The global effort to empower women's health through HPV vaccination programs is a dynamic and evolving landscape. The successes and challenges faced by different countries in achieving high vaccination coverage underscore the intricate interplay of socioeconomic factors, cultural considerations, and healthcare infrastructure. As we navigate this complex terrain, addressing economic disparities, understanding cultural nuances, and fortifying healthcare systems are integral to ensuring the widespread success of HPV vaccination initiatives on a global scale.

The objectives of the study are as follows: assess the global effectiveness of HPV vaccination programs in reducing cervical cancer rates across different countries; examine the influence of socio-economic and cultural factors on the success and challenges of HPV vaccination initiatives; and evaluate the impact of healthcare infrastructure on the implementation and sustainability of HPV vaccination programs.

## Review

Socio-economic factors influencing HPV vaccination success

This financial burden may discourage individuals from seeking vaccination, perpetuating disparities in coverage. To overcome these barriers, innovative strategies such as subsidy programs [5] can be implemented to reduce the economic strain on vulnerable populations. By mitigating financial obstacles, these strategies aim to enhance accessibility and contribute to the success of HPV vaccination initiatives [6].

Economic disparities play a pivotal role in determining access to HPV vaccines, consequently impacting the success of vaccination programs. Low-income populations often face hurdles in obtaining crucial healthcare services, including vaccinations. These economic disparities contribute significantly to the differential uptake of HPV vaccines, leading to increased vulnerability among disadvantaged groups [7]. This scenario underscores the urgent need for targeted interventions to address the economic aspects of HPV vaccination. Financial barriers pose a substantial challenge to widespread HPV vaccination. Families with limited financial resources encounter difficulties in affording the costs associated with vaccination, including consultation fees and vaccine doses [8].

Healthcare financing plays a critical role in determining the success of HPV vaccination coverage. The correlation between healthcare financing models and vaccination rates highlights how different funding structures can influence the accessibility and affordability of vaccines, ultimately affecting the overall success of vaccination programs [9]. Countries with comprehensive and accessible healthcare financing structures tend to exhibit higher vaccination coverage. Conversely, nations with fragmented or inequitable healthcare financing systems may witness disparities in HPV vaccine uptake. Recognising this, policymakers should prioritise the development and enhancement of healthcare financing mechanisms to ensure universal access to HPV vaccinations [10].

In addition to the economic dimension, cultural factors can influence the success of HPV vaccination programs. Cultural beliefs and practices may impact individuals' decisions to seek vaccination. In some communities, prevalent misconceptions, such as the belief that HPV vaccines promote promiscuity or that they cause infertility, along with cultural stigmas surrounding sexual health, may deter people from participating in vaccination programs [11]. Therefore, interventions should incorporate culturally sensitive approaches, such as engaging community leaders, providing education through trusted local sources, and tailoring messaging to respect cultural values, to comprehensively address the socioeconomic and cultural factors influencing vaccination success [12].

Regarding the effectiveness of preventive interventions, the study showed that educational programs and public-private cooperation enhance HPV vaccination. For example, traditional leaders can be used in publicity campaigns to persuade their followers to be vaccinated irrespective of any cultural beliefs. Also, budget outfits have promoted more extensive distribution of vaccines by optimising collaboration between government and private organisations offering health care. However, it stays restless where there is poor healthcare infrastructure; transportation challenges and cost can still be important determinants of a person’s ability to get the vaccines. Reducing the scope to socio-economic variables highlighted that income divide and access to healthcare are the major drivers of vaccination coverage, pointing to the requirements for targeted interventions that could help to level the differences.

**Figure 1:**
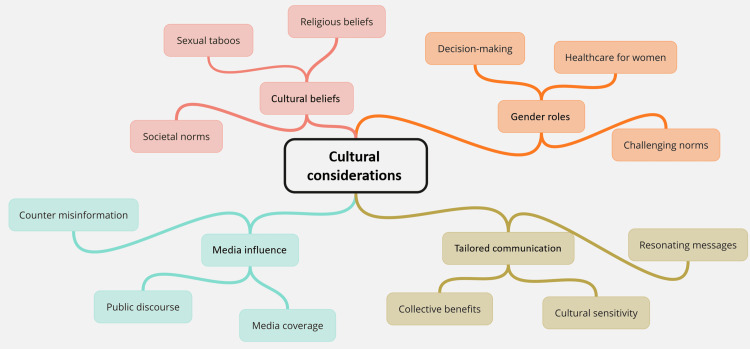
Socio-economic factors influencing human papillomavirus (HPV) vaccination success This image has been created by the author.

Furthermore, education and awareness campaigns are vital components of any successful vaccination program. Disseminating accurate information about HPV and the benefits of vaccination is crucial [13]. Improved education empowers individuals to make informed decisions, breaking down barriers rooted in misinformation or lack of awareness. To maximise the impact of these campaigns, collaboration between healthcare providers, community leaders, and educational institutions is essential (Figure 1) [14].

Cultural considerations and public perception of HPV vaccination

Cultural beliefs play a pivotal role in shaping the acceptance of HPV vaccines, significantly influencing public perception and uptake rates. Various cultural factors, such as religious beliefs and societal norms, contribute to the complex landscape of vaccine acceptance. For instance, cultural beliefs related to sexual taboos and conservative attitudes towards premarital sex in certain communities hindered the acceptance of HPV vaccination [15]. Addressing these cultural nuances is crucial for the success of vaccination campaigns. Moreover, understanding cultural beliefs is essential for tailoring communication strategies that resonate with specific communities [16].

Gender roles also impact the acceptance of HPV vaccines, reflecting societal norms surrounding healthcare decisions. In some cultures, men may play a pivotal role in decision-making regarding healthcare for women. Traditional gender roles can influence vaccination rates, as women's health decisions may be subject to male approval [17]. This underscores the importance of involving and educating both genders in HPV vaccination campaigns. Tailoring messages to challenge and reshape these gender norms is crucial to fostering a more inclusive and effective vaccination strategy.

Tailoring vaccination campaigns to diverse cultural contexts is imperative for breaking down barriers to HPV vaccine acceptance. Cultural sensitivity in communication materials and campaigns can significantly impact public perception. Tailoring messages to cultural values and beliefs increased the likelihood of vaccine acceptance [17]. This highlights the need for diverse and culturally competent approaches in public health campaigns. Emphasising the collective benefits of HPV vaccination while respecting cultural differences can contribute to increased trust and positive reception within various communities.

Public perception of HPV vaccination is not solely influenced by cultural beliefs but also by the broader societal context and public discourse. For instance, media coverage and public discussions can shape the narrative around vaccines. Media portrayal of HPV vaccination directly influenced public attitudes [18]. Negative portrayals and misinformation can significantly impact vaccination rates. Thus, a comprehensive approach that includes media engagement and education is necessary to counteract potential negative influences on public perception.

Cultural considerations and public perception are integral components of the success of HPV vaccination campaigns. Understanding and addressing cultural beliefs, navigating gender roles, and tailoring communication to diverse cultural contexts are essential for fostering acceptance. Additionally, recognising the broader societal influences on public perception, such as media representation, is crucial for crafting effective vaccination strategies. As efforts continue to empower women's health through HPV vaccination, a nuanced and culturally competent approach will be essential to ensure widespread acceptance and success.

Healthcare infrastructure and its role in HPV vaccination

Healthcare infrastructure plays a pivotal role in the success of vaccination programs, particularly in the context of preventing and combating diseases such as cervical cancer through HPV vaccination. The strength of healthcare systems is a crucial determinant in promoting vaccination initiatives. Robust healthcare systems with efficient delivery mechanisms contribute significantly to the success of vaccination programs [19]. Countries with well-established healthcare infrastructures are better equipped to implement and sustain HPV vaccination campaigns, ensuring widespread coverage and accessibility. Conversely, weaker healthcare systems may face challenges in reaching remote or underserved populations, limiting the overall impact of vaccination efforts [20].

Overcoming infrastructural challenges is essential to ensuring the effective distribution of HPV vaccines. In many regions, inadequate healthcare infrastructure poses hurdles to the seamless dissemination of vaccines. Logistic challenges, including insufficient storage facilities and transportation systems, can hinder the timely and widespread distribution of HPV vaccines [21]. To address this, collaborative efforts between governments, international organisations, and the private sector are imperative. The importance of public-private partnerships in enhancing healthcare infrastructure, thereby facilitating the successful implementation of vaccination programs [22]. These partnerships can contribute resources, expertise, and innovative solutions to overcome infrastructural barriers, ensuring that HPV vaccines reach the target populations efficiently.

Integrating vaccination programs into existing healthcare frameworks is a key strategy for optimising the impact of HPV vaccination on cervical cancer prevention. It is crucial to integrate vaccination initiatives into routine healthcare services [23]. By integrating HPV vaccination into existing healthcare frameworks, countries can capitalise on established channels for vaccine delivery, leveraging routine immunisation schedules and healthcare visits. This integration not only streamlines the vaccination process but also helps in normalising HPV vaccination as a routine preventive measure, reducing stigma and increasing acceptance among the target population. It requires collaborative efforts from policymakers, healthcare providers, and community stakeholders to seamlessly embed vaccination programs into broader healthcare services.

Despite the recognised importance of healthcare infrastructure, disparities in its development persist globally, affecting the equitable distribution of HPV vaccines. The World Health Organization (WHO) identifies the need for targeted interventions to address healthcare inequalities and ensure vulnerable populations have access to vaccination services [24,25]. Initiatives such as training healthcare professionals in remote areas, investing in mobile healthcare units, and leveraging technology for vaccine tracking and monitoring can contribute to reducing infrastructure-related disparities. By focusing on building healthcare infrastructure in marginalised regions, countries can create an environment conducive to the successful implementation of HPV vaccination programs, ultimately reducing the burden of cervical cancer [24-27].

The role of healthcare infrastructure in the context of HPV vaccination is multifaceted. The strength of healthcare systems determines the success of vaccination programs, with well-established infrastructure contributing to higher coverage and accessibility [28]. Overcoming infrastructural challenges is essential to ensuring the effective distribution of vaccines, requiring collaborative efforts and public-private partnerships. Integrating vaccination programs into existing healthcare frameworks is crucial for normalising HPV vaccination and optimising its impact. However, addressing disparities in healthcare infrastructure development is equally important to achieve equitable vaccine distribution. Policymakers, healthcare providers, and international organisations must work together to strengthen healthcare systems, overcome challenges, and integrate HPV vaccination seamlessly into broader healthcare services (Table 1).

**Table 1:** Key factors influencing human papillomavirus (HPV) vaccination success

Factor	Description	Examples
Strength of the healthcare infrastructure	Robust systems facilitate efficient vaccine delivery, contributing to higher coverage and accessibility.	Countries with advanced healthcare systems ensure widespread vaccination.
Infrastructural challenges	Inadequate infrastructure, such as insufficient storage facilities and transportation systems, hinders timely vaccine distribution.	Regions with poor logistics are facing delays in vaccine dissemination.
Public-private partnerships	Collaborative efforts between governments, international organisations, and private sectors enhance infrastructure, supporting the successful implementation.	Partnerships providing resources and expertise to overcome logistical barriers.
Integration with existing frameworks	Incorporating vaccination initiatives into routine healthcare services optimises impact and normalises HPV vaccination as a preventive measure.	Countries embedding HPV vaccination into routine immunisation schedules and healthcare visits.
Addressing infrastructure disparities	Targeted interventions to reduce inequalities in healthcare infrastructure, ensuring equitable vaccine distribution, particularly in marginalised regions.	Initiatives such as training healthcare workers in remote areas and using mobile healthcare units to reach vulnerable populations.

Factors affecting the HPV vaccination programme

Human papillomavirus vaccination is one of the most important preventive measures that can help avoid HPV and the diseases that it causes, including cervical cancer. However, HPV vaccination programs have been known to be affected by several socio-economic factors that determine the uptake of the vaccine among the various population groups.

Economic Disparities and Affordability

There is a close relationship between economic factors and the availability of HPV vaccines. The cost of vaccines and other related healthcare services may be a challenge, especially for the poor and those living in developing countries [29]. This is because the cost of vaccination does not only include the cost of the vaccine but also consultation fees and transportation costs to health facilities [29].

For instance, the price of HPV vaccines without insurance in the United States can exceed 16,400 Indian Rupees (₹) per dose, making the total cost over ₹49,200 for the complete series. This high cost can be prohibitively expensive for many families, particularly those with lower incomes, potentially leading to vaccine coverage rates of less than 50% among the less affluent [30]. The same issues are seen internationally; where cost is a barrier, there are fewer vaccinations, and health disparities continue [31].

Healthcare Financing Models

The nature of healthcare financing systems determines HPV vaccination coverage to a large extent. Healthcare systems that offer equal access to vaccines are more effective in delivering high vaccination rates than those with fragmented or inadequate health financing systems [32]. For example, in countries that have national immunisation programs that provide HPV vaccines at a subsidised or free of charge, as is the case in Australia and many European countries, the uptake of the vaccines is usually high [33].

On the other hand, countries where healthcare services are offered by private entities or where out-of-pocket expenses are necessary for vaccines end up with disparities in vaccination coverage, especially for vulnerable groups [34]. The fact that there is a relationship between healthcare financing and vaccination rates shows that equal access to healthcare services is crucial in promoting the uptake of vaccines [35].

Cultural Factors and HPV Vaccine Acceptance

The HPV vaccination acceptance is also determined by cultural beliefs and societal norms. Some cultural or religious groups may be reluctant to take vaccines, including HPV vaccines, due to misinformation they receive [36]. Sexual health culture, perception of the safety of vaccines, and perception of disease prevention influence vaccination decisions [37].

Some cultures may have a taboo against discussing or receiving vaccines for sexually transmitted infections (STIs) such as HPV. For example, only 45% of the public is aware that HPV is an STI, which can act as a barrier to youths and young adults seeking the vaccine despite its health benefits [38].

Education and Awareness Campaigns

Hence, education and awareness are critical components that need to be addressed to overcome the barriers to HPV vaccination. It is crucial to dispel myths and address concerns regarding HPV, cervical cancer, and vaccines, and this can only be done by providing the public with accurate information [39]. It also helps to increase the acceptance of vaccines when healthcare providers and other leaders in the community are educated and can be sources of information [40].

Countries that have implemented successful education campaigns, such as the United Kingdom with its HPV vaccine. A study has also shown that the promotion of the “Get the Facts: Help Protect Against Cervical Cancer” campaign has led to increased vaccine uptake among adolescents [41]. These campaigns focus on the need to get vaccinated early and also tackle myths that are related to HPV and its vaccines.

Healthcare Infrastructure and Vaccine Delivery

Healthcare system capacity is another important factor that determines the effectiveness of HPV vaccination programs. Healthcare systems that are already established and have proper means of delivering vaccines, such as proper storage and healthcare workers who can efficiently administer the vaccines, ensure that the vaccines are delivered in a timely and efficient manner [42].

**Figure 2:**
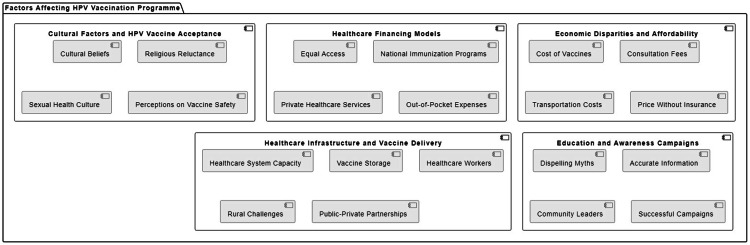
Factors affecting human papillomavirus (HPV) vaccination programs This image has been created by the author.

However, the scarcity of health facilities, especially in rural or hard-to-get areas, largely influences vaccine distribution and stock-outs. Solutions for this problem involve adding more vaccination mobile stations and enhancing the community’s healthcare system to consider rural areas [43]. For instance, leveraging partnerships with private institutions can augment the structures of vaccine conveyance and increase overall temperatures, which increases coverage rates [44]. Building the health infrastructure for delivering HPV vaccines is important for the success of HPV vaccination programs at different levels to have health equity (Figure 2) [45]. 

Methodologies of Drug Discovery in HPV 

Besides, the positive outcomes of vaccination prevention, the further developments in the field of traditional drug discovery approaches are significant for fighting HPV. Smaller molecules, including the molecules that can interact with viral proteins or host cell proteins, have been used to explore the possibility of preventing the infection of HPV as well as the replication of HPV. For example, some of the earlier compounds, such as cidofovir and other nucleotide derivatives, have been reported to exhibit activity in earlier studies [45]. Other strategies are also being developed, such as macromolecular ones, among them peptides. Some of the pharmaceuticals that have been developed can be used to prevent the virus from accessing host cells or the progression of the viral life cycle. For example, investigations into peptide vaccines are geared towards stimulating immune reactions that can target HPV proteins in a bid to improve the body’s affinity to fight the virus. These approaches are expected to add to traditional vaccination methods and create new ways through which HPV diseases can be treated [46].

## Conclusions

This paper proves that socio-economic determinants such as economic challenges in implementing the campaigns, accessibility, and costs, as well as cultural factors, have a direct impact on HPV vaccination across the world. Some of the research findings show that there are differences in economic status and the provisions for healthcare finances, as well as the cultural beliefs about immunisation, affecting the response in different demographics of people. To overcome these barriers, education and awareness of these barriers have to be provided together with the improved healthcare facilities that are needed for higher coverage of HPV vaccinations. It is important and should be recommended for women because the HPV vaccine significantly reduces the prevalence and also survival rates of cervical cancer. Thus, through the prevention of HPV infections, the vaccine does not only help decrease the sample adequacy control (SAC) across the countries’ healthcare systems but also the burden on patients and families. In addition, it supports gender equity through eradicating cervical cancer, which can easily be avoided; hence, women can act on their dreams without being derailed by a death sentence. However, there are barriers associated with the achievement of universal HPV vaccination coverage among the population, especially targeting minorities. To overcome these challenges, a complex strategy should be initiated, starting with increasing the population’s awareness of the problem and following healthcare policy changes. Combining HPV vaccination, cervical cancer screening, early detection, and sex-related practices makes the protective system of cervical cancer quite sound. By taking this approach of instituting vaccination, it is possible to not only reduce the spread of diseases among women but also ensure that inequality and poor support are contained. Addressing socio-economic, cultural, and infrastructural factors through targeted interventions and policies will enhance vaccination coverage and effectiveness, ultimately contributing to a significant reduction in cervical cancer incidence and improving overall women's health.
